# Impact of Targeted PPARγ Disruption on Bone Remodeling

**DOI:** 10.1016/j.mce.2015.01.045

**Published:** 2015-02-07

**Authors:** Jay Cao, Guomin Ou, Nianlan Yang, Kehong Ding, Barbara E. Kream, Mark W. Hamrick, Carlos M. Isales, Xing-Ming Shi

**Affiliations:** 1 USDA ARS Grand Forks Human Nutrition Research Center, Grand Forks, ND, USA; 2 Department of Neuroscience & Regenerative Medicine, Georgia Regents University, Augusta, GA, USA; 3 Department of Medicine, University of Connecticut Health Center, Farmington, CT, USA; 4 Department of Cell Biology, Georgia Regents University, Augusta, GA, USA; 5 Department of Orthopaedic Surgery, Georgia Regents University, Augusta, GA, USA

**Keywords:** PPARγ, MSC, aging, osteoblasts, bone loss

## Abstract

Peroxisome proliferator-activated receptor gamma (PPARγ), known as the master regulator of adipogenesis, has been regarded as a promising target for new anti-osteoporosis therapy due to its role in regulating bone marrow mesenchymal stem/progenitor cell (BMSC) lineage commitment. However, the precise mechanism underlying PPARγ regulation of bone is not clear as a bone-specific PPARγ conditional knockout (cKO) study has not been conducted and evidence showed that deletion of PPARγ in other tissues also have profound effect on bone. In this study, we show that mice deficiency of PPARγ in cells expressing a 3.6kb type I collagen promoter fragment (PPAR^fl/fl^:Col3.6-Cre) exhibit a moderate, site-dependent bone mass phenotype. *In vitro* studies showed that adipogenesis is abolished completely and osteoblastogenesis increased significantly in both primary bone marrow culture and the BMSCs isolated from PPARγ cKO mice. Histology and histomorphometry studies revealed significant increases in the numbers of osteoblasts and surface in the PPARγ cKO mice. Finally, we found that neither the differentiation nor the function of osteoclasts was affected in the PPARγ cKO mice. Together, our studies indicate that PPARγ plays an important role in bone remodeling by increasing the abundance of osteoblasts for repair, but not during skeletal development.

## 1. Introduction

Peroxisome proliferator-activated receptor gamma (PPARγ) is a ligand-activated nuclear receptor and is indispensible for adipocyte differentiation both *in vitro* and *in vivo* (1, 2). Bone marrow mesenchymal stem cell (BMSC) is a common precursor for bone-forming osteoblasts and marrow adipocytes. With advancing age, bone mass decreases and marrow fat increases (fatty marrow) (3-5), indicating that an imbalance develops between these two pathways (6-8). Many factors including the diet can influence the balance between these two pathways resulting in high marrow fat content and low bone density (9). Since the PPARγ is a key adipogenic regulator and the osteoblast and adipocyte, which are the two dominant pathways for BMSC to differentiate, have a reciprocal relationship (10-12), the PPARγ has been viewed as a prominent target for new anti-osteoporosis therapies that could increase bone formation or prevent bone loss. Indeed, studies have shown that PPARγ heterozygous (PPARγ^+/-^) mice exhibited an extraordinary high bone mass phenotype (13). However, global PPARγ insufficiency reduced body fat mass, therefore it is not clear whether the high bone mass observed in these PPARγ insufficient mice was due to a direct effect of PPARγ on BMSC lineage determination or an indirect effect via modulating the functions of adipose tissue or both, since disruption of PPARγ in adipose tissue (lipodystrophic PPARγ^hyp/hyp^ mice) also enhanced osteoblastic activity and increased bone formation (14). In this study, we generated mice in which PPARγ gene is deleted in cells that support the activity of a 3.6kb type I collagen promoter fragment (PPARγ^fl/fl^:Col3.6Cre) and report here the bone phenotype of these PPARγ conditional knockout (PPARγ cKO) mice.

## 2. Materials and Methods

### 2.1. Animals

PPARγ cKO mice were created by breeding Col3.6-Cre transgenic mice (15) with PPARγ floxed (PPARγ^fl/fl^) mice (16) using standard breeding procedures.

### 2.2. Ethics Statement

Animals were maintained in a centralized barrier facility and all experimental procedures were approved by the Institutional Animal Care and Use Committee (IACUC) at the Georgia Regents University.

### 2.3. Genotyping

Genotype was analyzed using primers and PCR conditions described previously (15, 17). In brief, tail genomic DNA was extracted and amplified with one forward primer (F1: 5′-CTCCAATGTTCTCAAACTTAC-3′) and two reverse primers (R1: 5′-GATGAGTCATGTAA GTTGACC-3′; R2: 5′-GTATTCTATGGCTTCCAGTGC-3′). The expected sizes of the PCR products are wild-type allele ∼250bp, floxed allele ∼285bp, and null allele ∼450bp. PCR analysis of Cre was performed using primers 5′-GCATTTCTGGGGATTGCTTA-3′ (forward) and 5′-GTCATCCTTAGCGCCGTAAA-3′ (reverse). The expected size of PCR product for Cre is ∼350 bp.

### 2.4. DXA and Micro Computed Tomography (μCT) analysis

Bone mineral density (BMD) and bone mineral content (BMC) were measured at 6, 12, and 30 weeks of age by dual-energy X-ray absorptiometry (DXA) (GE Lunar PIXImus system, software version 1.4×) as previously described (18). In brief, mice were anesthetized with a cocktail of ketamine hydrochloride and xylazine (30 mg/ml and 4 mg/ml, respectively, 0.1 ml/25 g body weight, IP) and were placed on the blocks supplied with the instrument according to the manufacturer's recommendations. All mice were analyzed at three distinct regions: (1) the total region, the whole body excluding the head; (2) a femoral region of interest that was defined as the maximal box fitted within a portion of the right femur; (3) a lumbar region of interest that was defined as the biggest box fitted within a portion of the lumbar vertebrae. The stability of the measurement was controlled by performing a quality control procedure before acquiring images. μCT scan (μCT-40; Scanco Medical AG, Bassersdorf, Switzerland) was used to evaluate the bone structural parameters as previously described (18).

### 2.5. Histology and histomorphometry assays

Bone tissues were collected, processed, and analyzed following our procedures described previously (19, 20). Standard bone histomorphometrical nomenclatures, symbols, and units were used as recommended by Parfitt et al (21).

#### 2.6. Primary bone marrow cell culture, isolation and differentiation of BMSCs

Bone marrow cells were flushed from long bones of PPARγ cKO, heterozygous, and PPARγ^fl/fl^ mice. Colony-forming unit (CFU) assays, BMSC isolation and differentiation were performed as previously described (22, 23).

### 2.7. In vitro osteoclast differentiation and bone resorption assay

Bone marrow monocyte and macrophage precursor cells (BMMs) were harvested from long bones and induced for osteoclast differentiation as described (18). In brief, BMMs were seeded in 96-well plates at a density of 1 × 10^4^ cells/well in triplicates and cultured in α-MEM containing FBS (10%), M-CSF (50 ng/ml) and RANKL (50 ng/ml) (R&D Systems, Inc. Minneapolis, MN) for 5-6 days. Differentiated cells were fixed, stained for tartrate-resistant acid phosphatase (TRAP) (#387A-1KT, Sigma Aldrich). For Pit assay, BMMs were seeded in coated quartz slides (Osteologic bonecell culture system, BD Biosciences) and induced for osteoclast differentiation as above. The slides were then stained with 5% silver nitrate solution (von Kossa staining) and the bone resorption was visualized as white spots (pits) on the slides.

### 2.8. Western blot analysis

Western blot analyses were performed as previously described (22, 24). Protein levels of PPARγ were detected using a monoclonal antibody raised against a sequence mapping at the C-terminus of PPARγ (Cat #: sc-7273, Santa Cruz Biotechnology, Inc.).

#### 2.9. Immunofluoresence labeling and Imaging

This experiment was performed as previously described (25) except that a PPARγ2 monoclonal antibody was used. Images were acquired using a Nikon TE2000 fluorescence microscope equipped with COOLSNAP Monochrome Camera and processed with Metamorph Imaging System.

### 2.10. Real-time qRT-PCR analysis

BMSC culture, RNA isolation, and quantitative analysis of levels of mRNA expression were performed as described previously using TaqMan Reverse Transcription Reagents (Applied Biosystems) and a Chromo-4 real-time RT-PCR instrument (MJ Research) (23). The mRNA levels were normalized to β-actin (internal control) and gene expression was presented as fold changes (ΔΔ Ct method). The primer sequences used in the PCR reactions are: Runx2: 5′-CCACCACTCACTACCACACG-3′ (forward) and 5′-TCAGCGTCAACACCATCATT-3′ (reverse); Colla1: 5′-CACCCTCAAGAGCCTGAGTC-3′ (forward) and 5′-CGGGCTGATGTACCAGTTCT-3′ (reverse); ALP: 5′-AACCCAGACACAAGCATTCC-3′ (forward) and 5′-CCAGCAAGAAGAAGCCTTTG-3′ (reverse); Ocn: 5′-TTCTGCTCACTCTGCTGACC-3′ (forward) and 5′-TTTGTAGGCGGTCTTCAAGC-3′ (reverse); PPARγ2: 5′-TTTTCCGAAGAACCATCCGAT-3′ (forward) and 5′-ACAAATGGTGATTTGTCCGTT-3′ (reverse); β-actin: 5′-CTGGCACCACACCTTCTACA-3′ (forward) and 5′-GGTACGACCAGAGGCATACA-3′ (reverse).

### 2.11. Statistical analysis

Results are expressed as mean ± SD. All in vitro experiments (qRT-PCR, cell differentiation etc.,) were performed in triplicates except where noted. Data were analyzed using either ANOVA with Bonferroni post hoc test or unpaired *t-test*, using a commercial statistical package (Instat, Graphad Inc.). A *p*-value less than 0.05 was considered significant.

## 3. Results

### 3.1. Bone phenotype of PPARγ cKO mice

#### Bone mineral density (BMD) and bone mineral content (BMC)

DXA scan was performed to detect dynamic changes of BMD and BMC. The measurement was started with mice at 6 weeks of age. This time point was based on studies by Akune et al (13), which showed that PPARγ haploinsufficient (PPARγ^+/-^) mice exhibit high bone mass at 8 weeks of age. We predicted that the PPARγ^fl/fl^:Col3.6-Cre mice would have a greater bone mass increase at an earlier time point than the PPARγ^+/-^ mice. However, we found no difference in BMD and BMC between cKO and floxed littermate control mice at 6 weeks of age either at the whole body or at the regional levels (data not shown). At 3 months of age, a moderate, yet statistically significant, increase in BMD and BMC (7.9% and 7.7%, respectively, cKO vs. flox) was detected in the lumbar spine of the male cKO mice. The magnitude of increase became more evident at 7.5 months (BMD 12%, BMC 10%, cKO vs. flox) (Table 1).

**Figure 1:**
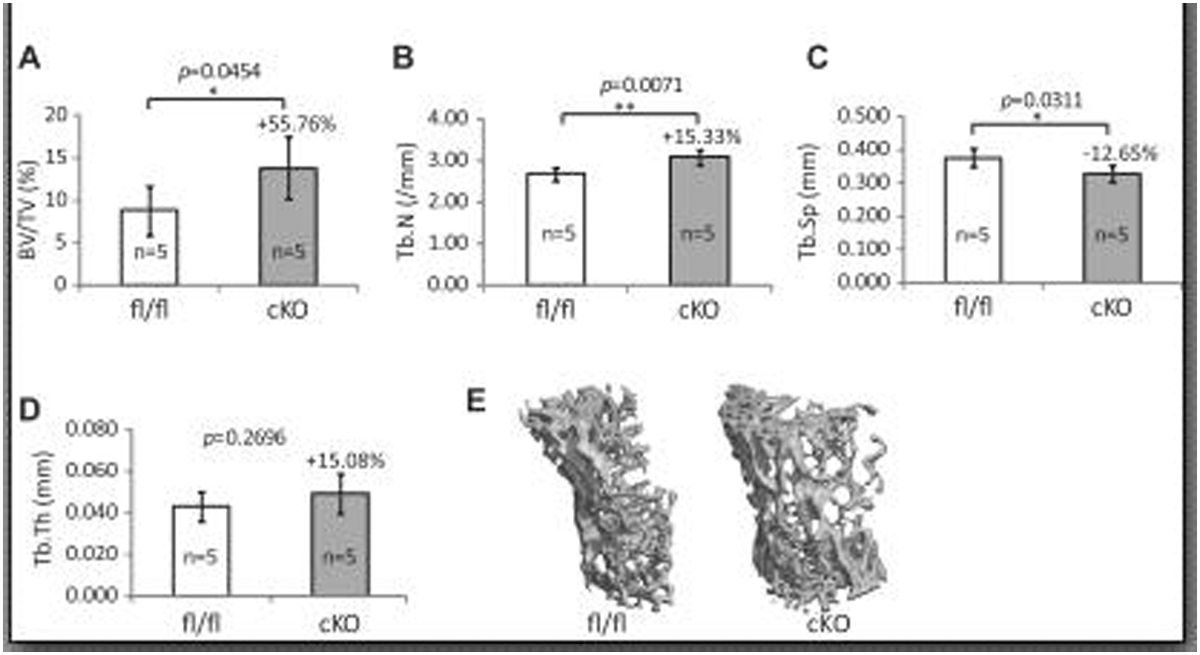
**(A-D)** *μ*CT analysis of lumbar spine **(**L4) showing BV/TV, Tb. N, Tb. Sp and Tb. Th of 6.5-month-old male PPARγ cKO (solid bar) and double-floxed control (empty bar) mice. **(E)** Representative three-dimensional reconstructed images of L4 lumbar spine. Values are given as mean ± SD.

μCT analysis of lumbar spine (L4, 6.5 months of age) showed that the bone volume density (BV/TV) and trabecular number (Tb. N) were increased (56% and 15%, respectively; *p* <0.05, n=5) and trabecular separation (Tb. Sp) decreased (-12%, *p* <0.05, n=5) significantly in PPARγ cKO mice compared to that in PPARγ^fl/fl^ control mice (Fig. 1A-C). The trabecular thickness (Tb. Th) showed an increase in cKO mice (+15%, *p*=0.270, n=5), although this increase is not statistically significant (Fig. 1D). Representative re-constructed 3D images are shown in Fig. 1E.

**Figure 2:**
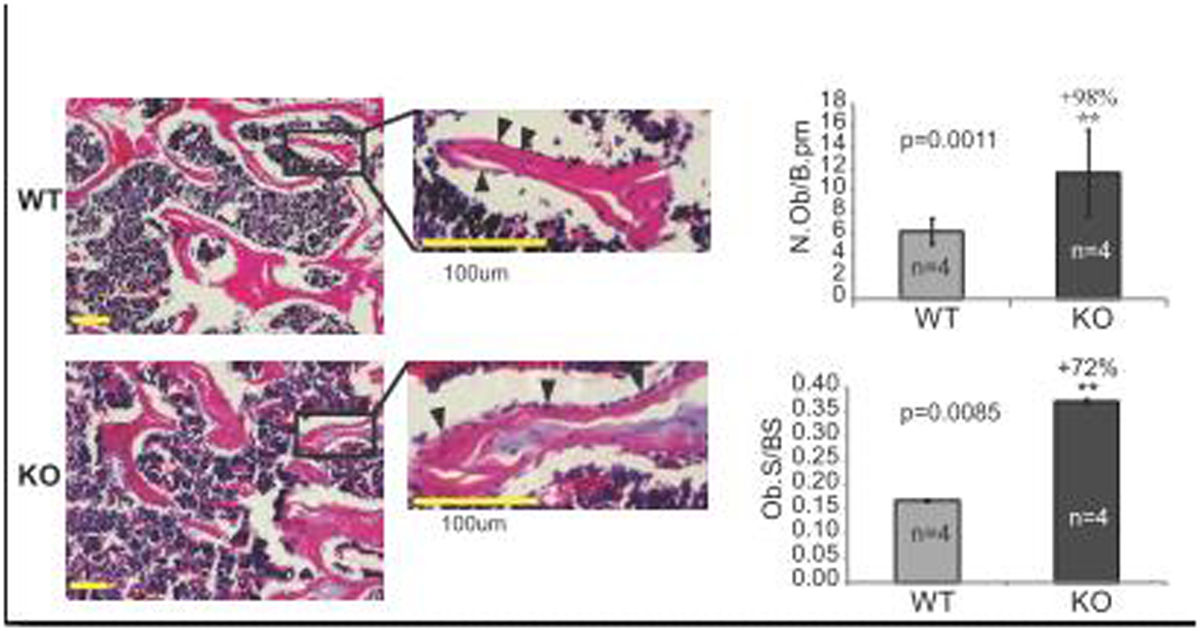
Histology and histomorphometry analyses **(A, B)** Representative H&E stained lumbar vertebrae (L4) from 6.5-month-old male mice. **(C, D)** Enlarged images showing the boxed areas in A and B. **(E, F)** Quantitative results showing the numbers of osteoblasts and the osteoblast covered bone surfaces.

Histology and histomorphometric analyses of lumbar spine (L2, 6.5 months of age) showed that both the number and the surface of osteoblasts were increased significantly in the PPARγ cKO mice compared to that in floxed control mice (+98% and +72%, respectively) (Fig. 2).

### 3.2. PPARγ gene is ablated successfully in BMSCs

**Figure 3:**
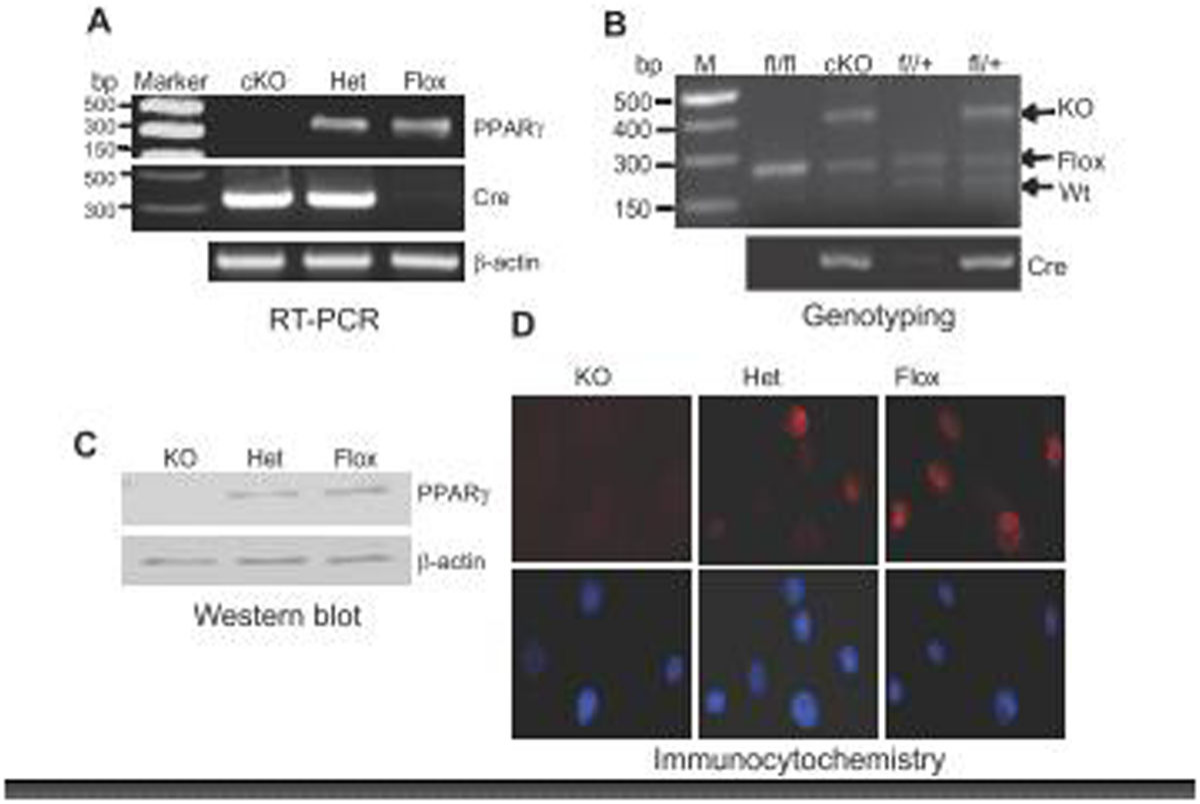
Characterization of BMSCs isolated from the PPARγ cKO mice **(A)** RT-PCR analysis showing the expression of PPARγ and Cre mRNA in BMSCs isolated from PPARγ cKO, heterozygous (Het), and Floxed (Flox) mice. **(B)** Genotyping results showing distinct PCR products amplified from tail genomic DNA. KO and floxed PPARγ alleles are indicated. WT: tail DNA from a wild-type mouse was used as template in PCR reaction to show the wild type allele. **(C, D)** Western blot (C) and immunofluorescence labeling (D) showing PPARγ protein expression in BMSCs.

Since an expected high bone mass phenotype was not observed in the PPARγ cKO mice, we suspected that the Col3.6kb promoter fragment was probably not active in BMSCs but only in a small fraction of committed osteoblasts. To clarify this conjecture, we isolated BMSCs using a negative-immuno-depletion and positive-immuno-selection procedure established in our laboratory (23) and examined the expression of PPARγ mRNA. RT-PCR analysis showed that the PPARγ mRNA is, indeed, absent in the BMSCs of cKO mice while its expression detected in cells of PPARγ-flox and heterozygous mice (Fig. 3A). The Cre mRNA, which is present in cells of heterozygous and cKO mice, is absent in cells of double floxed mice. These results are consistent with the genotyping results obtained from PCR amplification of the tail genomic DNA (Fig. 3B). To confirm the deletion of PPARγ at the protein level, we performed Western blot and immunocytochemistry studies using a monoclonal antibody specific to PPARγ. Results of both experiments revealed, again, that the PPARγ protein is absent in cells isolated from cKO mice, but expressed in cells of heterozygous and PPARγ-flox mice (Fig. 3C, D). These results demonstrated that the Col3.6kb promoter fragment is active, robustly drives Cre expression, and ablates PPARγ allele in BMSCs.

### 3.3. Adipogenic differentiation is abolished in PPARγ cKO cell cultures *in vitro*

**Figure 4:**
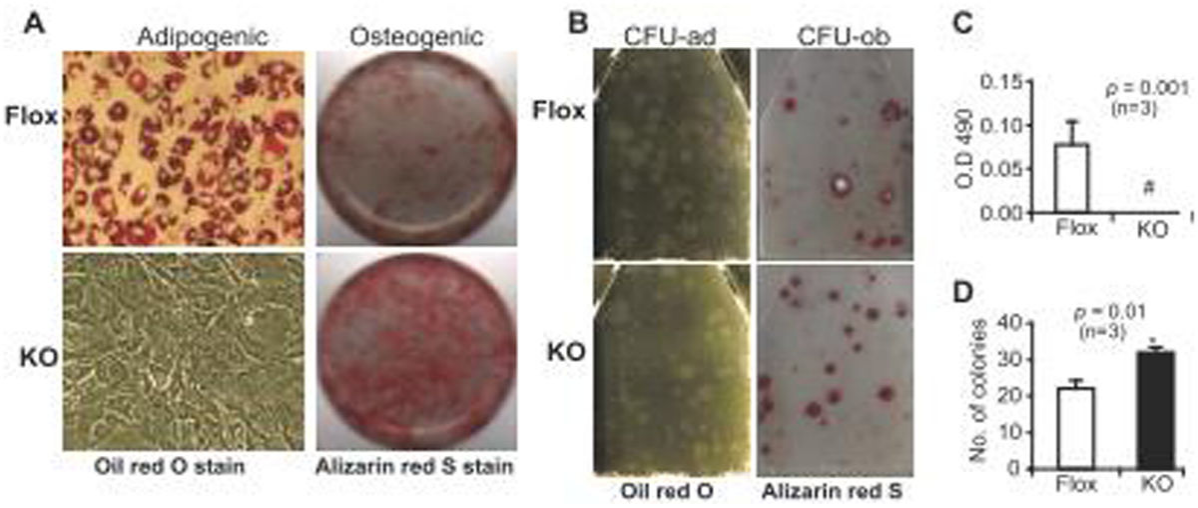
Adipogenic and osteogenic differentiation assays **(A)** BMSCs were subjected to standard adipocyte or osteoblast differentiation program. Cells were either stained with Oil red O for detection of adipocytes 9 days after induction (A, left panel), or stained with Alizarin red S for detection of mineralized bone nodules 21 days after induction (A, right panel). **(B)** Assays for the number of colony forming units (CFUs). Equal numbers of bone marrow cells (5×10^6^ cells/25cm^2^ flask) from cKO and double-floxed mice were cultured in adipogenic or osteogenic induction media and stained with Oil Red O at day 11 for CFU-ad or with Alizarin red S at day 21 for CFU-ob. Colonies (>50 cells in size) were counted visually. These experiments were repeated two times in triplicates. Only one representative flask is shown for each group. **(C, D)** Quantitative results of B.

To demonstrate, at the functional level, that PPARγ gene is deleted in BMSCs, we subjected BMSCs to adipogenic and osteogenic differentiation program. Fig. 4 shows that while the PPARγ-flox BMSCs differentiated into adipocytes normally, as determined by Oil Red O staining of the intracellular lipid droplets, the PPARγ KO cells completely lost their ability for adipocyte differentiation (Fig. 4A). As expected, in vitro osteogenic differentiation of PPARγ KO BMSC is enhanced significantly as assessed by Alizarin red S (ARS) staining of the mineralized bone nodules (Fig. 4A). This result is replicated in colony forming unit (CFU) assays using freshly prepared whole bone marrow cells, i.e., CFU-adipocyte (CFU-ad) is completely abolished and CFU-osteoblast (CFU-ob) enhanced significantly in marrow cell cultures of PPARγ cKO mice (Fig. 4B). Quantitative results of these assays are shown in Fig. 4C and D. These results are in line with the RT-PCR and western blot and immunocytochemistry studies. Together, these data demonstrated that the PPARγ gene is deleted by Col3.6kb promoter-driven Cre in BMSCs or progenitor cells that are capable of differentiating into at least two cell lineages, the adipocytes and osteoblasts.

### 3.4. Expression of osteoblast lineage-associated genes in PPARγ KO BMSCs

**Figure 5:**
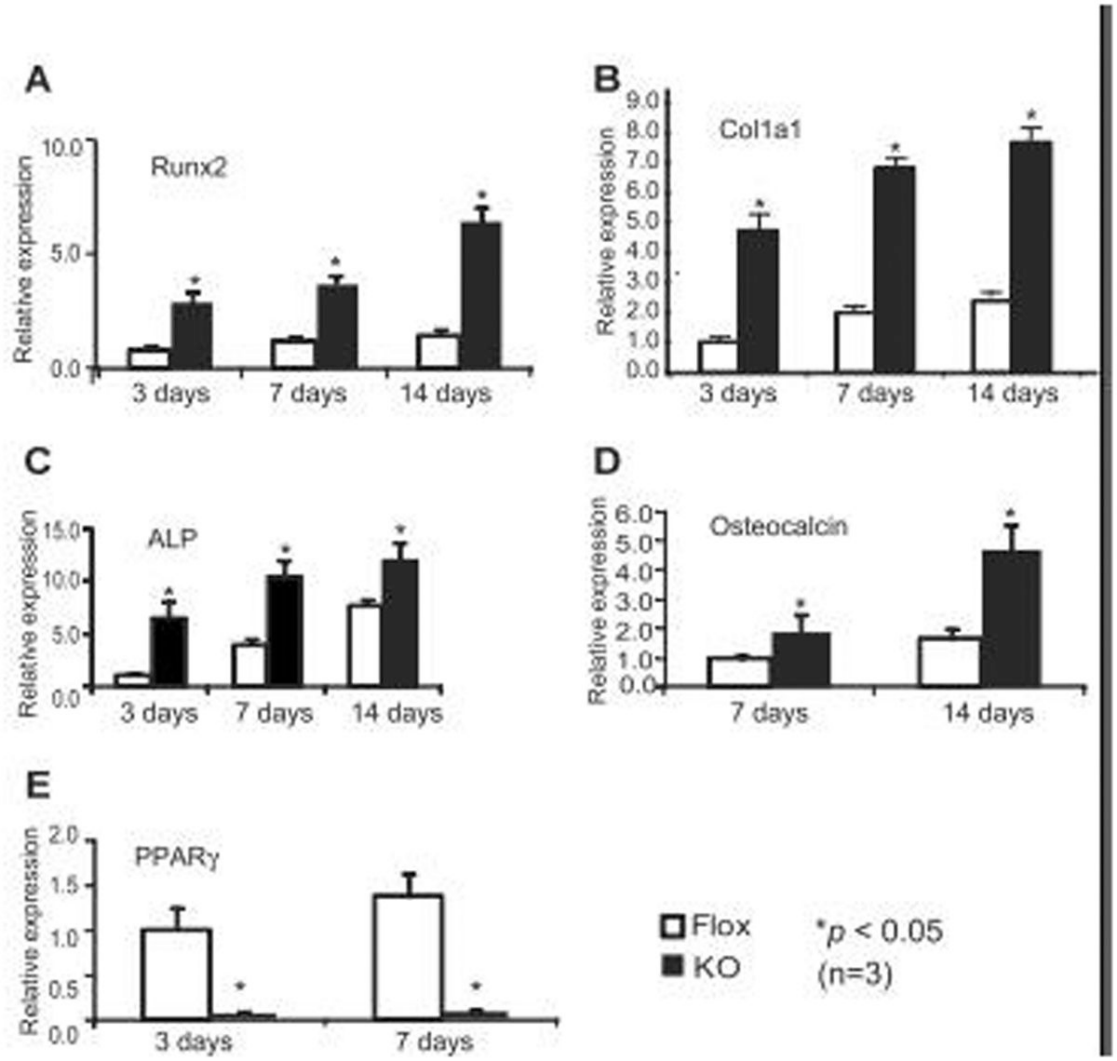
Real-time qRT-PCR analysis of mRNA expression during BMSC osteogenic differentiation BMSCs were cultured in osteogenic induction media and harvested at days 3, 7, and 14 for total RNA isolation. Equal amounts of RNA were reverse transcribed and the mRNA levels of the indicated genes were analyzed. Data were normalized to β-actin and expressed as fold changes. Levels of mRNA in double-floxed BMSCs at day 3 were arbitrarily set as 1. Experiments were repeated two times. PCR reactions were performed in triplicates. Error bars indicate SD. *p < 0.05.

To determine if the observed increase in osteogenesis of PPARγ KO cells correlates with the expression pattern of osteoblast lineage genes, we performed real-time qRT-PCR analysis. BMSCs from PPARγ cKO and floxed mice were treated with osteogenic induction media and analyzed for the expression of mRNAs. Results showed that the mRNA levels of Runx2 (Fig. 5A), type I collagen (5B), alkaline phosphatase (5C), and osteocalcin (5D) were all increased significantly in PPARγ KO cells compared to that in PPARγ-floxed cells. Levels of PPARγ mRNA in these cells are also shown (5E). These results demonstrated that BMSCs lacking PPARγ do have an enhanced *in vitro* osteogenic differentiation capability.

### 3.5. KO of PPARγ in BMSCs does not affect osteoclast differentiation and function

**Figure 6:**
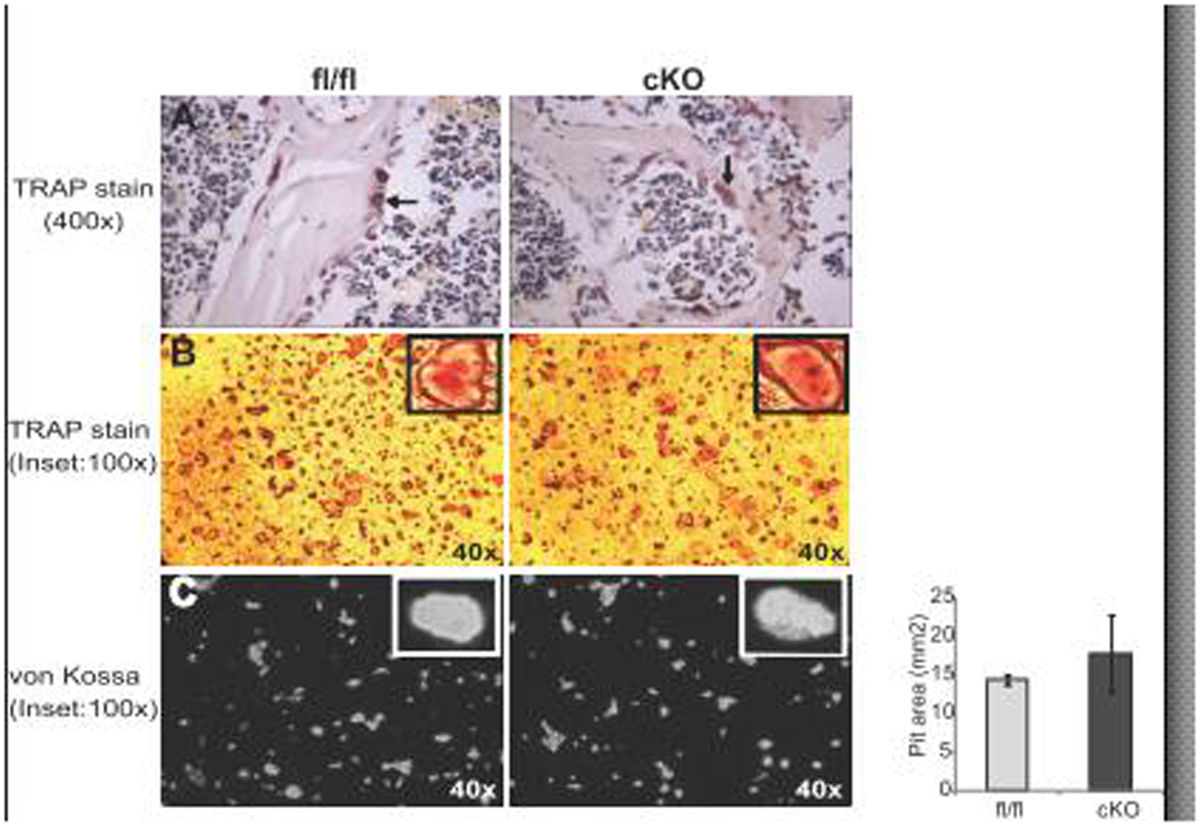
Effect of PPARγ on osteoclast differentiation and function **(A)** TRAP stain of decalcified tibiae samples showing the TRAP-positive osteoclasts in PPARγ cKO and double-floxed control mice at 7 months of age. **(B)** BMMs isolated from PPARγ cKO and floxed mice were induced for osteoclast differentiation in the presence of M-CSF (50ng/ml) and RANKL (50ng/ml) for 6 days and stained for TRAP activity. **(C)** Pit assay showing bone resorption activity. BMMs were induced as above in quartz chamber slides and stained with 5% silver nitrate. White spots indicate the area where bone resorption occurred. Insets: representative high power images of osteoclasts and pits shown in A and B. Original magnification of the images including insets is indicated. These experiments were performed at least 3 times and with different concentrations of RANKL, same results were obtained.

Lastly, we examined osteoclastogenesis both *in vivo*, using decalcified tibia samples, and *in vitro*, using bone marrow monocyte/macrophage precursor cells (BMMs). TRAP stain experiment showed that neither the numbers of osteoclasts (N.Oc/B.Pm) nor the osteoclast surfaces (N.Oc/BS) were different between PPARγ cKO and floxed control mice (Fig. 6A). *In vitro* osteoclast induction experiment showed that BMMs from PPARγ cKO and floxed mice differentiated into multi-nucleated TRAP-positive osteoclasts equally well (Fig. 6B), demonstrating that the osteoclast differentiation is not affected. To determine whether these osteoclasts are functionally different, we performed a bone resorption assay. BMMs were seeded in coated quartz slides and induced for osteoclast differentiation. The slides were then fixed and stained with 5% silver nitrate solution (von Kossa staining) to assess the bone resorption activity. Again, the results showed that the osteoclasts differentiated from BMMs of cKO and floxed control mice had similar bone resorption activity (Fig. 6C). These results suggested that deletion of PPARγ in osteoblastic lineage cells or BMSCs does not have an effect on osteoclast differentiation or function. This conclusion is consistent to and supported, in some degree, by studies of Akune et al, which showed that the osteoclastogenesis is not affected in PPARγ-haploinsufficient mice (13).

## 4. Discussion

In this study we showed that deletion of PPARγ gene in 3.6kb type I collagen promoter-expressing cells (PPAR^fl/fl^:Col3.6-Cre) resulted in a moderately enhanced bone mineral density preferentially in the vertebrae of the elder mice (Table 1). Multiple experiments, from DNA to protein expression showed clearly that the PPARγ gene is deleted in cells supporting the activities of Col3.6kb promoter fragment (Fig. 3). Giving the high expectations for the impact of PPARγ knockout may have on bone, and the fact that the adipogenesis of both primary bone marrow cells and the BMSCs isolated from the PPARγ cKO mice is abolished completely (Fig. 4), it is a little disappointing that the bone density increase was not dramatic in the PPAR^fl/fl^:Col3.6-Cre mice. Interestingly, however, a similar bone phenotype was also observed in another conditional PPARγ knockout mouse model, the PPARγ^fl/fl^:Sox2-Cre mouse (26). This mouse, which carries total deletion of PPARγ, showed an increased BMD only in vertebrae but not in long bones (26). This phenotype is similar to the leptin-deficient ob/ob mouse, which shows increased bone mass in the spine and decreased bone mass in the limb (19). More recently, Sun et al reported that osteoblast-specific PPARγ KO (at ∼3 weeks of age) using an inducible Osx promoter-driven Cre (PPARγ^fl/fl^:Sp7-tTA,tetO-EGFP/Cre) increased the Tb. N and decreased Tb. Sp in 6-month-old mice, but had no effect on BMD of the femur (long bone) and the authors concluded that suppression of PPARγ increases osteogenesis through activation of mTOR signaling (27). These results clearly indicated that other mechanisms by which PPARγ regulates bone exist. A plausible one, as proposed by Gimble and colleagues (28) and Ferrari and colleagues (26), in addition to mTOR pathway, is that PPARγ regulates bone turnover/remodeling by both a direct effect on cell differentiation and an indirect effect on cell fate determination through adipocyte and adipokine secretion (26). Supporting this notion, studies of lipodystrophic PPARγ^hyp/hyp^ mouse, which lacks PPARγ only in adipose tissues, also showed increased osteogenic activity and enhanced bone formation (14). The adipose tissue is an endocrine organ and secretes large amounts of factors including adipokines and inflammatory cytokines that are known to regulate bone formation and metabolism (29-41). Currently, it is not clear, and controversial, whether PPARγ plays a role in osteoclast differentiation or function. Our studies showed that the osteoclast differentiation was not affected in PPAR^fl/fl^:Col3.6-Cre cKO mice (Fig. 6). Studies by Akune et al showed that osteoclast differentiation and function are not affected in PPARγ-haploinsufficient mice (13). Interestingly, Wan et al showed that mice lacking PPARγ in osteoclast lineage cells (Tie2-Cre:PPAR^f/f^) develop severe osteopetrosis, suggesting that PPARγ is a strong pro-osteoclast factor (42). In contrast, several lines of *in vitro* evidence showed that activation of PPARγ by its ligands inhibits osteoclast differentiation (43-50). However, it is not clear whether this ligand-mediated inhibition is PPARγ dependent or independent.

Bone is a dynamic enterprise; it is constantly destroyed by osteoclasts and rebuilt by osteoblasts in a process called bone remodeling. Bone remodeling occurs constantly throughout the developed skeleton and is a key process maintaining bone homeostasis. While the activity of resorption and formation may keep in balance when bone mass is at its peak level or shortly after, during the phase of plateau, this balance is broken in old human or animals with more bones destroyed than new bones formed, thus generates a deficit in each remodeling cycle due to the lack of osteoblasts for repair (34, 51). Bone loss with aging is accompanied by an increase in marrow fat content (3-5), and this coincide with the increase of PPARγ (52-54). Our data that knockout PPARγ in BMSC/progenitor cells increased the numbers of osteoblasts suggests that PPARγ may play an important role in aging-induced bone loss, i.e., as the bone marrow microenvironment deteriorates with advancing age, the levels of PPARγ expression and/or activity increase. These changes lead to an increased marrow adipogenesis and the production of inflammatory cytokines and adipokines, which impact negatively on the production of osteoblasts and consequently, the bone repair during bone remodeling.

## Figures and Tables

**Table 1:** BMD and BMC of male PPARγ cKO and fl/fl mice at 3 and 7.5 months of age

Age	3 month			7.5 month		
Genotype	cKO (n=12)	fl/fl (n=8)	*p*	cKO (n=13)	fl/fl (n=13)	*p*
**Whole body**						
BMD (g/cm^2^)	0.0536±0.0021	0.0526±0.0014	0.2820	0.0593±0.0031	0.0556±0.0021	**0.0017**
BMC (g)	0.534±0.047	0.526±0.038	0.6749	0.639±0.063	0.574±0.045	**0.0053**
**Spine**						
BMD (g/cm^2^)	0.0618±0.0031	0.0573±0.0035	**0.0067**	0.0664±0.0054	0.0594±0.0052	**0.0026**
BMC (g)	0.042±0.002	0.039±0.003	**0.0423**	0.044±0.005	0.040±0.006	**0.0407**
**Femur**						
BMD (g/cm^2^)	0.0772±0.0060	0.0775±0.0023	0.9019	0.0855±0.0077	0.0809±0.0053	0.0880
BMC (g)	0.034±0.003	0.033±0.002	0.7735	0.038±0.005	0.036±0.003	0.1053

## Highlights

- A bone marrow mesenchymal stem/progenitor cell specific PPARγ conditional knockout mouse model was generated using a 3.6kb type I collagen promoter-driven Cre line.
- The PPARγ cKO mice exhibited a moderate, site-dependent bone mass phenotype.
- The *In vitro* adipogenesis was abolished completely in bone marrow cells of PPARγ cKO mice.
- The differentiation and function of osteoclasts were not affected in t PPARγ cKO mice.
